# A Systems Biology and LASSO-Based Approach to Decipher the Transcriptome–Interactome Signature for Predicting Non-Small Cell Lung Cancer

**DOI:** 10.3390/biology11121752

**Published:** 2022-11-30

**Authors:** Firoz Ahmed, Abdul Arif Khan, Hifzur Rahman Ansari, Absarul Haque

**Affiliations:** 1 Department of Biochemistry, College of Science, University of Jeddah, P.O. Box 80327, Jeddah 21589, Saudi Arabia; 2 Department of Pharmaceutics, College of Pharmacy, King Saud University, P.O. Box 2457, Riyadh 11451, Saudi Arabia; 3 King Abdullah International Medical Research Center (KAIMRC), King Saud Bin Abdulaziz University for Health Sciences, King Abdulaziz Medical City, Ministry of National Guard Health Affairs, P.O. Box 9515, Jeddah 21423, Saudi Arabia; 4 King Fahd Medical Research Center, King Abdulaziz University, P.O. Box 80216, Jeddah 21589, Saudi Arabia; 5 Department of Medical Laboratory Sciences, Faculty of Applied Medical Sciences, King Abdulaziz University, P.O. Box 80216, Jeddah 21589, Saudi Arabia

**Keywords:** non-small cell lung cancer, LASSO model, artificial intelligence, gene expression, biological networks, hub genes

## Abstract

**Simple Summary:**

Non-small cell lung cancer (NSCLC) is a serious public health issue due to its high mortality rate. To improve the survival rate of NSCLC with better treatment, it is imperative to develop a biomarker-based prediction tool that can accurately identify NSCLC at a very early stage. Cancer development initiates due to aberrations in gene expression and the regulatory networks; therefore, these features hold a great potential to diagnose cancer at an early stage compared with the visible morphological and pathological changes. In this study, we integrated gene expression and interactome data to identify candidate genes altered in NSCLC compared with normal samples. We then used a machine learning technique to identify a signature of 17 genes and developed a model for predicting NSCLC. Interestingly, our model predicted NSCLC across different independent test datasets with high accuracy. Finally, the model was implemented to create a user-friendly web tool, *NSCLCpred*, to predict NSCLC using the expression profile of 17 genes. We expect that our findings will guide the identification of NSCLC patients and provide more insight into the understanding of disease development.

**Abstract:**

The lack of precise molecular signatures limits the early diagnosis of non-small cell lung cancer (NSCLC). The present study used gene expression data and interaction networks to develop a highly accurate model with the least absolute shrinkage and selection operator (LASSO) for predicting NSCLC. The differentially expressed genes (DEGs) were identified in NSCLC compared with normal tissues using TCGA and GTEx data. A biological network was constructed using DEGs, and the top 20 upregulated and 20 downregulated hub genes were identified. These hub genes were used to identify signature genes with penalized logistic regression using the LASSO to predict NSCLC. Our model’s development involved the following steps: (i) the dataset was divided into 80% for training (TR) and 20% for testing (TD1); (ii) a LASSO logistic regression analysis was performed on the TR with 10-fold cross-validation and identified a combination of 17 genes as NSCLC predictors, which were used further for development of the LASSO model. The model’s performance was assessed on the TD1 dataset and achieved an accuracy and an area under the curve of the receiver operating characteristics (AUC-ROC) of 0.986 and 0.998, respectively. Furthermore, the performance of the LASSO model was evaluated using three independent NSCLC test datasets (GSE18842, GSE27262, GSE19804) and achieved high accuracy, with an AUC-ROC of >0.99, >0.99, and 0.95, respectively. Based on this study, a web application called *NSCLCpred* was developed to predict NSCLC.

## 1. Introduction

Despite all the rapid advancements in the development of anticancer therapy, lung cancer is still a significant contributor to cancer-associated deaths, with almost 1.8 million worldwide mortalities recorded in the year 2020 [1]. Non-small cell lung cancer (NSCLC) is a major contributor to lung cancer cases, making up almost 85% of cases of primary lung cancer [2]. The high mortality rate associated with NSCLC is because the disease is often diagnosed late in most patients, resulting in a poor prognosis, even with the availability of advanced treatment modalities [3]. Furthermore, nearly half of the initially diagnosed early-stage tumors (Stage I or II) in patients eventually proceed to the late stage, resulting in metastatic NSCLC. Advanced NSCLC is generally categorized into Stage IIIB or IV tumors, and its current treatments options include immunotherapy, systemic chemotherapy, and targeted drug therapy, which often lead to imposing remarkably great adverse impacts not only on the lives of patients but also at the socio-economic level [4,5,6], especially the family and friends providing informal care to the NSCLC patient. The continuous increase in the economic burden has posed a great financial challenge to society, as the number of advanced NSCLC cases is increasing. Thus, it appears that the patients and their caregivers seem to be affected by the due course of stage progression. As a result, it directly influences the increase in direct and indirect costs as the stages of the disease advance. It has been shown that the overall economic burden of the management of lung cancer in Europe is considerably surprising because the direct costs of caring for such NSCLC patients amount to more than EUR 3 billion annually [7]. As is quite evident from several studies, the clinical outcome for NSCLC patients is directly dependent on the stage of the tumor when it is diagnosed [8,9]. So far, screening for NSCLC patients relies on using chest radiographs or sputum cytologic profile analyses, which remain adequate and have failed to provide a mortality benefit in many studies of clinical trials [8,9]. Therefore, there is a need to focus on discovering and validating a set of biomarkers with high sensitivity and specific discriminatory power that might be utilized in early screening programs along with having diagnostic and prognostic significance that would allow the accurate detection of such patients in the early stages of the disease, consequently enabling the clinician to reduce the mortality rate of NSCLC patients.

However, imaging techniques, including X-ray, CT, MRI, PET scans, and tissue biopsy, are routine practices in diagnosing lung cancer and are generally used when the cancer is at an advanced stage [10]. The powerful imaging techniques can detect a tumor only when its size is at least 7 mm with billions of cells [11]. Unfortunately, only 16% of cases are detected before the spread of lung cancer to other organs [12]. However, the recent advancement of high-throughput sequencing technology has improved the understanding of the underlying pathological changes and identified the genomic and environmental factors involved in lung cancer [13,14]. The accumulated knowledge is being utilized for advancing diagnostic accuracy, and a significant improvement has been gained in the treatment outcomes in several cases. In addition to these technological improvements, evidence has indicated that utilizing a growing number of molecular-level approaches could be beneficial for improving the early diagnosis and treatment outcomes of lung cancer. Hence, there is a great need to adopt advanced molecular techniques for early diagnosis, better management, and treatment of cancer patients in super-specialized hospitals [15]. In the past few decades, studies have used different approaches to identify the genes and mutation signatures underpinning lung cancer, leading to new therapeutic targets for better treatment. A previous study used an integrative systems biology approach and revealed a driver network that promotes cell proliferation in NSCLC, which could be a promising therapeutic target [16]. Interestingly, the study found that the driver network consisted of 26 upregulated genes associated with spindles, kinetochores, nuclear division, chromosome segregation, and the cell cycle G2/M transition and their upstream regulators, FOXM1 and MYBL2 [16]. A recent study used gene expression data from the TGF-β-induced epithelial–mesenchymal transition in NSCLC cells and identified a cluster of differentially expressed genes associated with specific metabolic processes such as glycolysis, pyruvate metabolism, and the tricarboxylic acid cycle [17]. Interestingly, the same study elucidated the potential links in the regulation of NSCLC’s progression and found 10 genes as prognostic biomarkers associated with a decrease in the overall survival of NSCLC patients. The prognostic markers might be helpful for evaluating treatment outcomes and monitoring and selecting suitable therapeutic strategies in NSCLC [17]. Integrated bioinformatic analysis of differentially expressed genes was successfully used to identify the potential prognostic gene signatures in other cancers, including esophageal squamous cell carcinoma and cervical cancer [18,19]. 

Furthermore, several models have been developed for predicting the risk score for lung cancer [20,21,22,23]. However, these studies mainly focused on utilizing epidemiological factors, symptoms, and clinical assessments as features, not gene expression features, for development of the model; therefore, these models have been suggested to have certain limitations due to their low accuracy. Another study developed a prognostic model for NSCLC patients using immune-specific transcriptomic and clinicopathological data, and achieved an area under the curve (AUC) of 0.673 [24]. Failure to diagnose lung cancer at an early stage, resulting in metastasis to other organs, has posed a significant challenge in treating cancer thoroughly. Therefore, an early-stage detection method with the highest possible accuracy is essential for better treatment and prognosis of NSCLC. 

The recent advancement and breakthroughs in high-throughput sequencing technology have enabled the rapid growth of transcriptomic and other omics data from cancer samples, thus providing an excellent opportunity to improve deep insights and early diagnosis [25,26]. However, identifying the signature genes for early cancer diagnoses and the interpretation of the underlying mechanisms in high-dimensional and complex data remains a great challenge [27,28]. Artificial intelligence (AI) and machine learning techniques (MLT) have successfully been used in biomedicine and crop improvement [29,30,31,32,33]. The application of AI and MLT has also shown promising results in cancer diagnosis and drug discovery, where a predictive model can be built by learning and generalizing from the training data. The model is applied to new data to make predictions [34,35,36].

This work combined transcriptome–interactome signatures to develop an efficient model for predicting NSCLC. Briefly, we used the gene expression profile to identify the differentially expressed genes (DEGs), followed by finding the hub genes in biological networks. These hub genes were used to select the signature genes that best discriminated NSCLC from normal samples by the least absolute shrinkage and selection operator (LASSO). The highly accurate predictive LASSO model was developed by using selected features, and then the results were interpreted for a mechanical understanding. In feature selection, LASSO penalizes the regression variable coefficient and shrinks them to zero. After that, it selects the variables with non-zero coefficients for constructing the model. The larger the parameter λ, the greater the number of coefficients that shrink to zero. Therefore, we have to tune and select the minimum value of the parameter λ to obtain a sufficient number of coefficients. The present work demonstrated the potential use of this approach for developing predictive models for the early diagnosis of other cancers.

## 2. Materials and Methods

**Figure 1:**
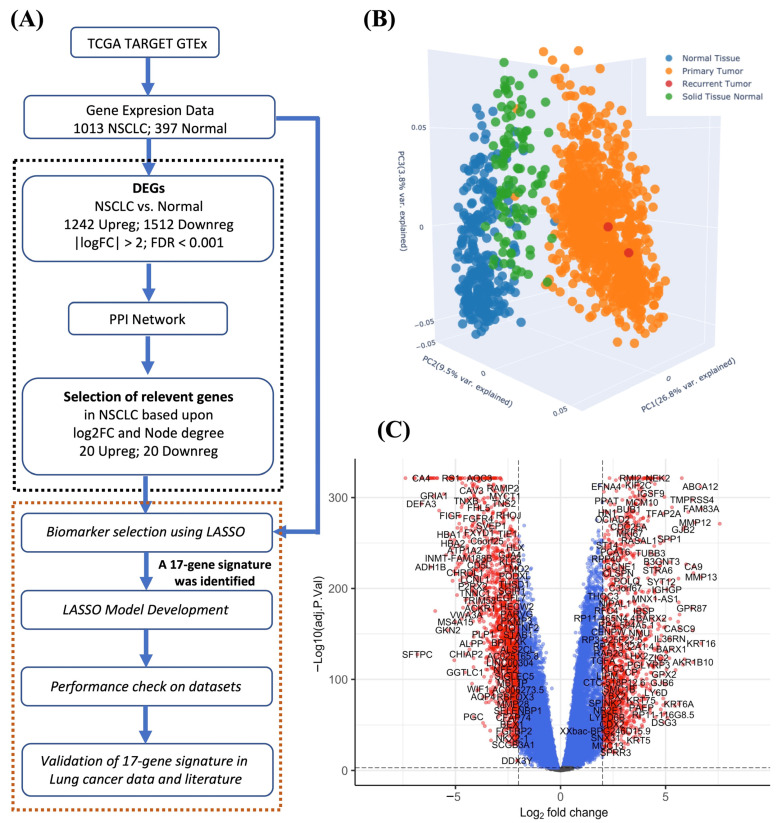
(**A**) Workflow of our study. (**B**) The PCA plot for samples using 2500 genes with the most significant variance. Each point represents the gene expression of a sample. Samples with similar gene expression profiles are closer in the three-dimensional space. (**C**) Volcano plot of DEGs in lung cancer compared with normal samples. The DEGs with |log2FC| > 2.0 and adj.p.val < 0.001 are shown in red.

The experimental workflow of our study is given in Figure 1A and consisted of two parts. The first part of the work identified the biologically important genes associated with NSCLC using DEGs and their interaction network. The next part used the relevant genes, identified in the first part of the work as input for feature selection and model development with the LASSO. Finally, the model’s performance was evaluated on independent test datasets and the 17-gene signature was validated in lung cancer data and literature.

### 2.1. Identification of DEGs

The gene expression data of the NSCLC and normal samples were obtained from TCGA TARGET GTEx using the UCSC Xena application (https://xena.ucsc.edu/, accessed on 7 August 2021). The gene expression dataset was in “RSEM norm_count” format. The NSCLC data consisted of 1013 samples of lung adenocarcinoma and lung squamous cell carcinoma taken from The Cancer Genome Atlas (TCGA) [37]. The normal sample included 397 samples of “lung solid tissue—normal” collected from TCGA and “normal lung tissue” taken from the Genotype-Tissue Expression (GTEx) database [38]. TCGA provides gene expression and other omics data as well as clinical data from primary cancer and matched normal samples across 33 cancer types. GTEx provides tissue-specific gene expression and regulation data from nearly 1000 non-diseased individuals. The DEGs in lung cancer compared with normal samples were analyzed using the Xena application adapted from the Appyter RNA-seq analysis pipeline from Ma’ayan lab (https://github.com/MaayanLab/appyter-catalog, accessed on 7 August 2021). The RNA-seq data underwent quantile normalization, and the DEGs were identified. A gene was considered to be upregulated when log2FC > 2 and adj.p.value < 0.001, but deemed to be downregulated when log2FC < −2 and adj.p.value < 0.001. The volcano plot was made using the *EnhancedVolcano* tool in R version 4.1.2 [39].

### 2.2. Construction of the Interaction Network

The biological interactions data of humans were screened from BioGRID version 4.4.205 (last modified 29 December 2021) [40]. The interaction data were filtered to screen the interactions of the identified DEGs in lung cancer. The DEGs data were added to the interaction network, and the sub-network was prepared on the basis of the log2FC value. Cytoscape version 3.8 was used to visualize the interaction network of biologically important genes [41]. 

### 2.3. Identification of Biologically Important Nodes in the Network

The network topology was calculated using Cytoscape’s built-in *NetworkAnalyzer* tool. The nodes’ sizes were arranged according to the value of their degree in the original BioGRID human interaction network. In contrast, the color of the nodes was set as per their log2FC value in cancer and normal samples in the lung. The interaction network was further filtered based on the log2FC value in lung samples. 

### 2.4. Training and Testing Dataset

The final dataset consisted of the expression values in the RSEM of 40 genes identified by analyzing the DEGs and interactomes from 1013 samples of lung cancer and 397 samples as the control. This dataset was divided into two parts: an 80% training dataset (TR, with 1128 samples) and a 20% test dataset 1 (TD1, with 282 samples). The model was developed using 10-fold cross-validation (cv) on the TR dataset, and the performance was checked on TD1 dataset (Table 1). To further validate the accuracy and robustness of the LASSO model, we used additional validation test dataset 2 (TD2), which contained the gene expression data of lung cancer obtained from three microarrays: GSE18842 [42], GSE27262 [43], and GSE19804 [44]. These raw microarray data were normalized using the RMA of the *Oligo package* in R. The number of samples of lung cancer and the adjacent non-tumor tissues in TD2 is provided in Table S1.

### 2.5. Construction of the LASSO Model

We used the R package *glmnet* version 4.1-3 to develop a penalized logistic regression LASSO model on the TR dataset with 10-fold cv. We divided the data randomly into 10 sets; of these, we used 9 sets for training and the remaining set for testing. First, the penalty regularization parameter lambda was determined by 10-fold cv with the *cv.glmnet* module. Next, the final model was developed by *glmnet* with a lambda value which maximized the value of AUC (lambda.min). The expression data of genes with non-zero coefficients were used to create the final LASSO model. 

### 2.6. Performance of the Models

The performance of the LASSO models was evaluated with the following parameters.

(1) *Sensitivity*, also called the recall or true positive rate, which indicates the percentage of correctly predicted cancer samples.

$$Sensitivity=\frac{TP}{TP+FN}$$

(2) *Specificity*, which indicates the percentage of correctly predicted normal samples.

$$Specificity=\frac{TN}{TN+FP}$$

(3) *Accuracy* is the percentage of correct predictions overall.

$$Accuracy=\frac{TP+TN}{TP+FP+TN+FN}$$

(4) *Positive predictive value (PPV)*, also called the precision.

$$PPV=\frac{TP}{TP+FP}$$

(5) *Negative predictive value (NPV)*

$$NPV=\frac{TN}{TN+FN}$$

where *TP* stands for true positive, *TN* stands for true negative, *FP* stands for false positive, *FN* stands for false negative.

(6) *Area Under the Curve (AUC)*. The performance was tested at various thresholds using the receiver operating characteristics (ROC) to plot a graph of the true positive rate (sensitivity on the *y*-axis) versus the false positive rate (1 – specificity on the *x*-axis). The higher the mean AUC-ROC values, the better the model was for distinguishing between lung cancer and normal samples. In addition, we used precision–recall (PRC), which is a plot of the precision (positive predictive value on the *y*-axis) versus the recall (sensitivity or true positive rate on the *x*-axis) for all possible thresholds. The larger the value of AUC-PRC, the better the model’s performance. If the positive and negative data were imbalanced, the PRC curve was preferred for checking the model’s performance.

### 2.7. Functional Enrichment of Key Genes Obtained by the LASSO Model

Lung cancer signature genes identified by the LASSO were used to construct a separate interaction network of nodes and their first neighbors as per BioGRID. The signature genes were further analyzed for functional enrichment using Gene Ontology (biological processes) and for pathway enrichment using KEGG with DAVID version 6.8 [45].

## 3. Results

### 3.1. Identification of DEGs

In order to find the genes associated with lung cancer, we performed a differential gene expression analysis using the limma-voom tool [46]. The expression profiles of the 2500 genes with the greatest variance were used for PCA analysis. Three principal components comprising 40.1% (26.8%, 9.5%, and 3.8%) of the total variance showed that the lung cancer samples were clustered apart from the normal samples (Figure 1B). Furthermore, we obtained 2754 DEGs, including 1242 upregulated and 1512 downregulated genes in lung cancer compared with normal samples with |log2FC| > 2, and adj.p.value < 0.001 (Figure 1C). According to the log2FC values, the top five upregulated genes were *CST1, FAM83A, KRT16, MMP13*, and *MMP12*, whereas the top five downregulated genes were *DEFA1B, SLC6A4, DEFA1, SFTPC*, and *CA4* (Table S2). The complete list of upregulated and downregulated genes is provided in Supplementary Tables S3 and S4, respectively. 

### 3.2. Identification of the Relevant Interacting Genes

**Figure 2:**
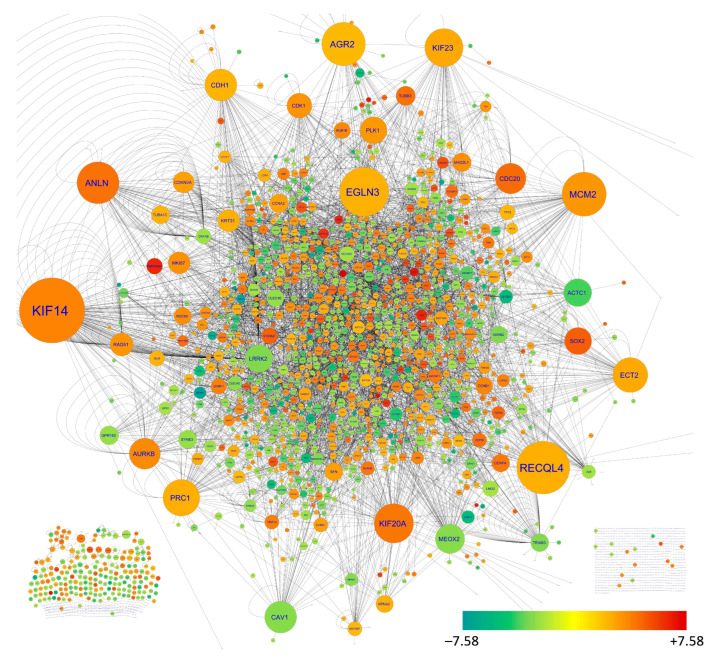
Biological interaction network of DEGs identified in NSCLC samples according to BioGRID v 4.4.205. The DEGs’ nodes were filtered from all interactions available in BioGRID. The node sizes are arranged as per their degree in the original human interaction network and therefore indicate their central involvement in human cellular interactions. The colors of the nodes were determined by their log2FC value, where green to red represents negative to positive log2FC values.

Human-related biological interaction data were obtained from BioGRID, which contains 40,843 nodes and 977,146 edges. This data in BioGRID also included some interactions involving species other than humans; therefore, the human-specific interactions were filtered, and we obtained 33,235 nodes with 909,098 edges. The network parameters, including the degree of the nodes, were calculated for this interaction network. The DEGs data were integrated into this interaction network, and the sub-network was filtered, as presented in Figure 2. The top 20 nodes according to the degree were further sorted on the basis of their log2FC value, thus revealing the top 20 upregulated hub genes and the top 20 downregulated hub genes (Table 2). 

### 3.3. Development of the LASSO Model

**Figure 3:**
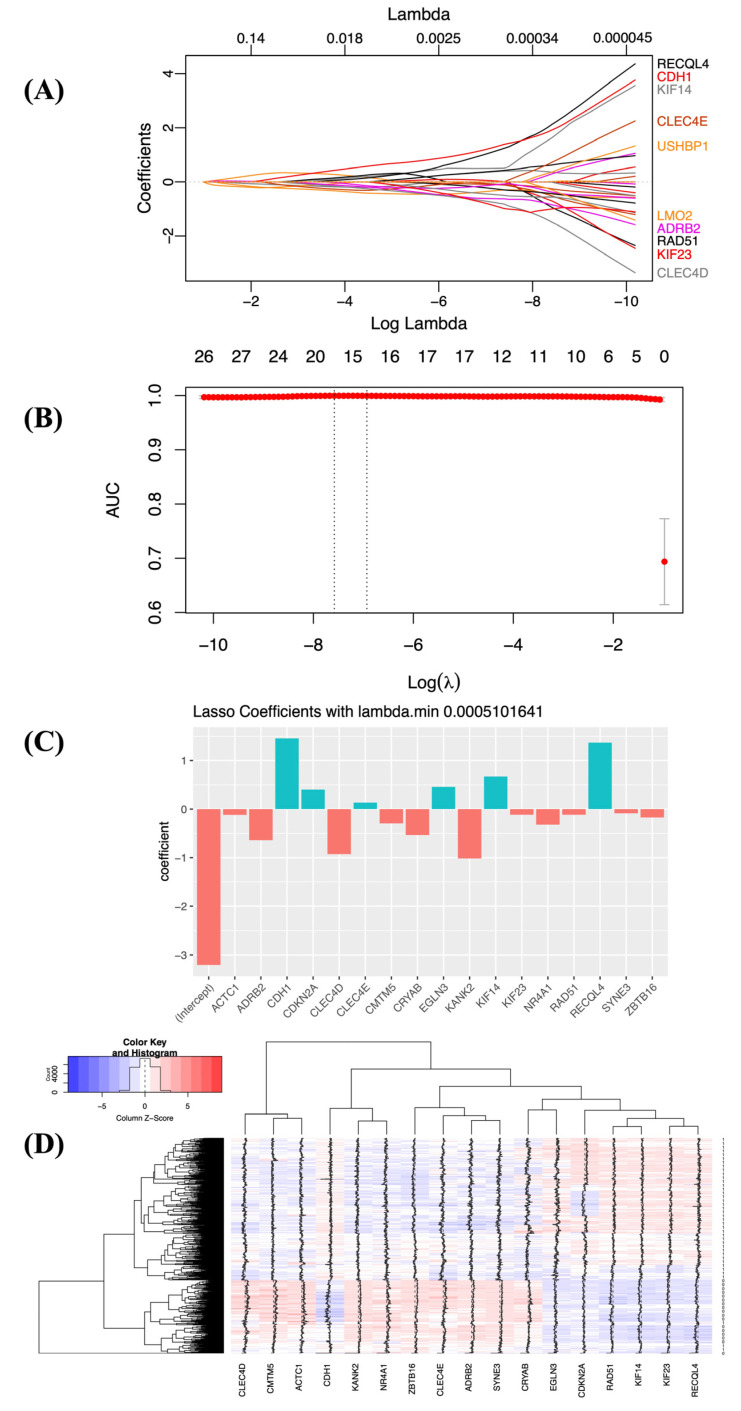
Construction of the risk score model for lung cancer prediction using LASSO logistic regression with 10-fold cv using *glmnet*. (**A**) LASSO regression coefficient profiles of 40 genes associated with lung cancer at different values of log lambda. Each curve indicates a gene and the path of its coefficient against the different values of log lambda. (**B**) This plot displays the AUC value (in red) with varying values of log lambda. The vertical dotted line at the left indicates the value of λ lambda.min that gives the maximum average AUC. The vertical dotted line at the right shows the largest value of λ lambda.1se; the performance is within one standard error of the maximum average AUC. The numbers across the top are the nonzero coefficient estimates. (**C**) Bar graph representing the regression coefficients for the most relevant genes (17 genes) at lambda.min = 0.0005101641. The blue-green bar represents positive coefficients; the red bar represents negative coefficients. (**D**) Heatmap of the expression patterns of relevant genes (17 genes) from the TR dataset.

The gene expression profiles of 40 hub genes in lung cancer and control samples were used to build a classifier in order to predict lung cancer. First, we fitted the LASSO logistic regression model and plotted the coefficients at different log lambda values (Figure 3A). The plot displays the behavior of coefficients at different values of lambda. After that, we used 10-fold cv to find the best value of lambda that maximized the AUC curve (Figure 3B). We selected lambda.min (0.0005101641) as the best lambda and identified the 17 important genes with non-zero coefficients (Figure 3C, Table S5). Furthermore, the gene expression patterns of these 17 important genes were extracted from the TR dataset, and a heatmap was plotted, revealing that their expression patterns in lung cancer and the normal sample were distinct (Figure 3D). The set of 17 genes was further explored to find the gene family in the Molecular Signatures Database (MsigDB v7.5.1: http://www.gsea-msigdb.org/gsea/msigdb/index.jsp, accessed on 15 June 2022) [47,48]. Based upon the protein homology or biochemical activity, we found that the dysregulated genes belonged to tumor suppressors, oncogenes, translocated cancer genes, and transcription factors (Table S6). Finally, the LASSO model was developed by using the expression profiles of 17 genes in the TR dataset at lambda.min (0.0005101641) for predicting lung cancer. The lung cancer signature genes in the LASSO model were present in the order of *KIF14, RAD51, CDKN2A, KIF23, RECQL4, EGLN3, CDH1, ZBTB16, CMTM5, ACTC1, ADRB2, NR4A1, CLEC4D, CLEC4E, SYNE3, CRYAB,* and *KANK2.* The model was constructed using the expression values of the 17 genes and their coefficients, and the risk score for lung cancer was calculated as follows.

Risk score for NSCLC = −3.207 + (−1.016 × KANK2) + (−0.929 × CLEC4D) + (−0.64 × ADRB2) +

(−0.533 × CRYAB) + (−0.322 × NR4A1) + (−0.297 × CMTM5) + (−0.174 × ZBTB16) +

(−0.12 × ACTC1) + (−0.118 × RAD51) + (−0.117 × KIF23) + (−0.087 × SYNE3) +

(0.136 × CLEC4E) + (0.403 × CDKN2A) + (0.459 × EGLN3) + (0.675 × KIF14) +

(1.372 × RECQL4) + (1.457 × CDH1)

where the gene name indicates its expression value in “RSEM norm_count”. A gene is associated with a lower risk of lung cancer if its coefficient is less than zero (0). On the contrary, a gene is associated with a higher risk of lung cancer if its coefficient is greater than zero (0).

### 3.4. Performance of the LASSO Model on Independent Datasets

**Figure 4:**
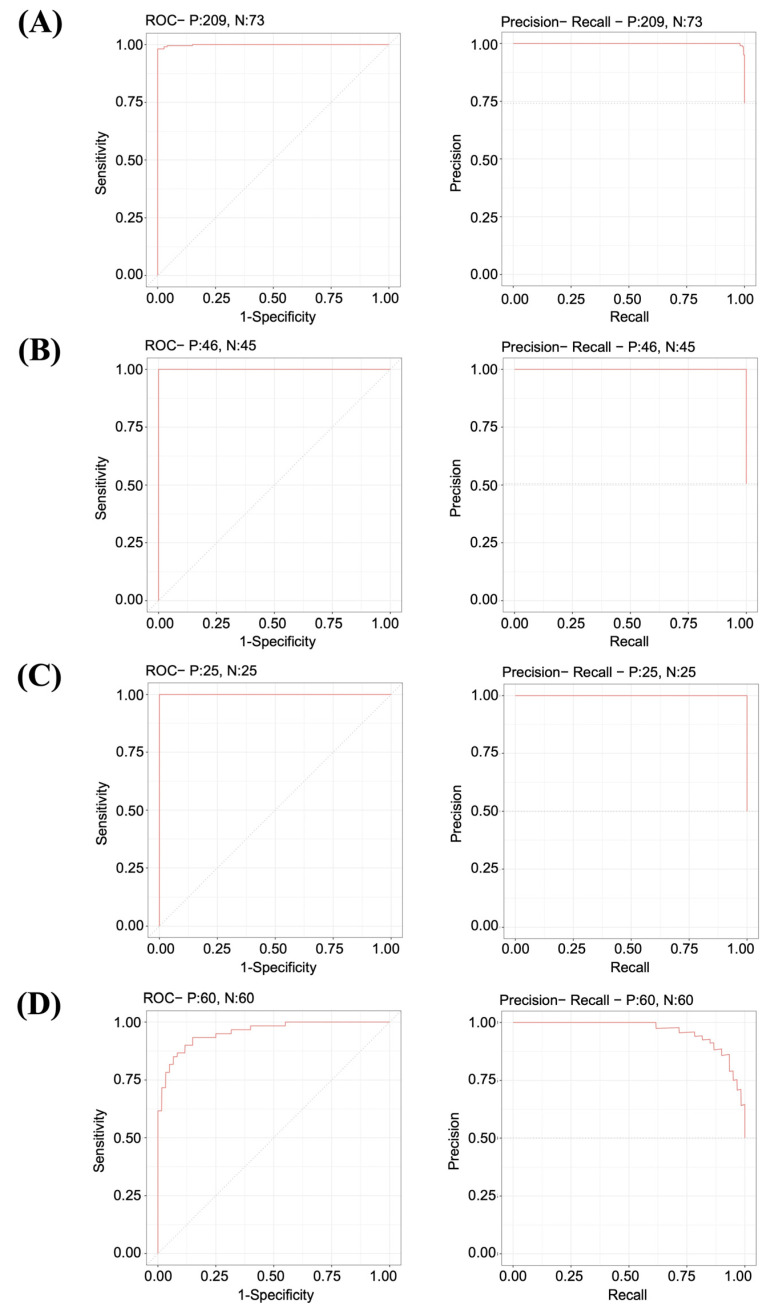
Performance of the LASSO model on the independent test datasets. (**A**) Performance on the TD1 dataset that contained 209 cancer and 73 normal samples, with the ROC curve showing an AUC of 0.9988 and the PRC curve showing an AUC of 0.999. (**B**) Performance on the GSE18842 dataset that contained 46 cancer and 45 normal samples, with the ROC curve showing an AUC of >0.99 and the PRC curve showing an AUC of >0.99. (**C**) Performance on the GSE27262 dataset that contained 25 cancer and 25 normal samples, with the ROC curve showing an AUC of >0.99 and the PRC curve showing an AUC of >0.99. (**D**) Performance on the GSE19804 dataset that contained 60 cancer and 60 normal samples, with the ROC curve showing an AUC of 0.95 and the PRC curve showing an AUC of 0.96. The ROC graphs plot the true positive rate (sensitivity on the *y*-axis) versus the false positive rate (1-specificity on the *x*-axis) for all possible thresholds. The value of the AUC varies from 0 to 1. The larger the value of the AUC, the better the model can differentiate between lung cancer and normal samples. The diagonal dashed line represents an AUC of 0.5, which indicates random prediction by the model. The PRC plots the precision (positive predictive value on the *y*-axis) versus the recall (sensitivity or true positive rate on the *x*-axis) for all possible thresholds. The larger the AUC, the better the model’s performance. The ROC and PRC curves were built with the R package *precrec*.

We evaluated the reliability of the LASSO model on the TD1 dataset, and found that the model achieved an accuracy, specificity, and sensitivity of 0.986, 0.959, and 0.995, respectively, at the 0.5 threshold (Table 3). The performance of the model showed an AUC-ROC and AUC-PRC of 0.9988 and 0.999, respectively, on the TD1 dataset (Figure 4A). Furthermore, we evaluated its performance on the TD2 dataset (GSE18842, GSE27262, and GSE19804) containing the gene expression patterns of NSCLC. On GSE18842, the LASSO model achieved an accuracy, specificity, and sensitivity of 1, 1, and 1, respectively, at the 0.5 threshold (Table S7). Furthermore, on the same data, the model showed an AUC-ROC and AUC-PRC of >0.99 and >0.99, respectively (Figure 4B). However, the performance of the LASSO model on GSE27262 achieved an accuracy, specificity, and sensitivity of 0.980, 1, and 0.960, respectively, at the 0.5 threshold (Table S8). Notably, the same model achieved 100% accuracy, specificity, and sensitivity when the threshold value was decreased to 0.4 as compared with 0.5 in the GSE27262 dataset (Table S8). The model showed an AUC-ROC and AUC-PRC of >0.99 and >0.99, respectively, on GSE27262 (Figure 4C). However, on the dataset of GSE19804, the model’s performance was reduced, having an accuracy, AUC-ROC, and AUC-PRC of 0.725, 0.95, and 0.96, respectively (Figure 4D and Table S9). 

### 3.5. Comparative Analysis of Logistic Regression Models

Furthermore, we also examined the performance of models developed via logistic regression using *glm* from the R package. The logistic regression models were developed on the TR dataset, and their performance was assessed using the test dataset TD1. We found that the logistic regression model developed from 40 genes identified on the basis of the log2FC and node degree achieved an AUC-ROC of 0.9828. In comparison, the performance was slightly reduced (AUC-ROC: 0.9789) when the model was developed by using the 17-gene signature (Figure S1). On the basis of the ROC curve, we concluded that the signatures of the 17 genes achieved better performance with the LASSO model (AUC: 0.9988) compared with logistic regression (AUC: 9789). The LASSO regression selected the important features by shrinking the coefficient towards zero, which also had the advantage of avoiding model overfitting, and interpreting the possible roles of the features in lung cancer. 

### 3.6. Interaction Network and Functional Enrichment Analysis of Genes from the LASSO Model

**Figure 5:**
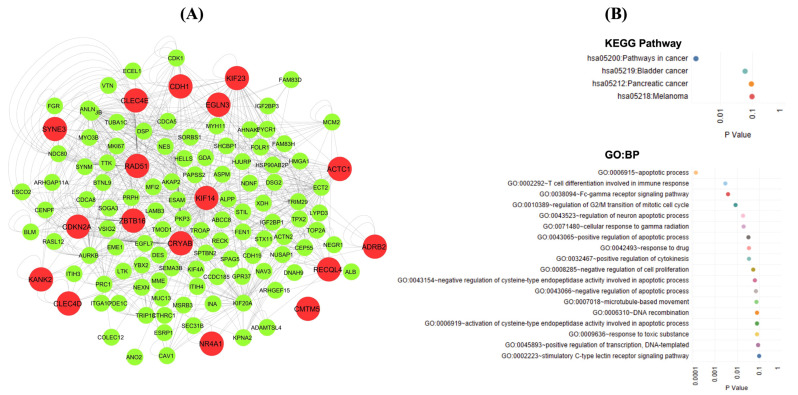
Sub-network of the signature genes and their functional enrichment identified through the LASSO. (**A**) Interaction network of genes identified through transcriptome–interactome signatures and their first neighbors. The genes are represented as red nodes, while their first neighbors are shown in green. (**B**) Functional enrichment analysis of 17 targets against the pathway database KEGG and biological process in the Gene Ontology database.

To understand the biological function of the signature genes, we performed a functional enrichment analysis with the DAVID bioinformatics tool (version 6.8). Figure 5A represents the interaction network of the nodes and their first neighbors. Functional enrichment analysis of 17 genes revealed that the signature genes were significantly involved in the cancer pathway and apoptotic process (Figure 5B). 

### 3.7. Validation of the 17-Gene Signature in Lung Cancer Data

We validated our identified 17-gene signature for NSCLC in various experimental studies using the Expression Atlas (release 38; https://www.ebi.ac.uk/gxa/home, accessed on 7 August 2022). We took the gene name, selected *Homo sapiens* as the species, and lung cancer as the biological condition, and submitted these to the database. Next, the differential expression data were downloaded with “diseases” as the experimental variables, and were considered only the data with a comparison between cancer vs. normal with |log2FC| > 2. The result identified that 14 genes were differentially expressed out of 17 across 32 studies (Table S10). We found positive LASSO coefficients for the genes *CDKN2A, EGLN3, KIF14*, and *RECQL4* that were upregulated in cancer compared with normal samples. On the contrary, negative LASSO coefficients were found for the genes *ADRB2, CRYAB, NR4A1, CMTM5, ZBTB16, SYNE3*, and *RAD51*, which were downregulated in cancer compared with normal samples. Negative LASSO coefficients were found for the genes KANK2 and CLEC4D that were downregulated in cancer compared with normal samples, but their log2FC was between −1 and −2. Thus, the results above support the efficient selection of genes by the LASSO for predicting NSCLC. However, we also found positive LASSO coefficients for the genes *CLEC4E*, and CDH1 that were downregulated, and a negative LASSO coefficient for the gene *KIF23*, which was both upregulated and downregulated in different studies.

## 4. Discussion

Detecting cancer at its early stage is the foremost goal of preventing cancer’s proliferation and metastasis. Therefore, developing highly accurate and reliable molecular diagnostic tools for cancer, including predictive and prognostic models, is indispensable for diagnosis at the early stage and finding suitable treatment modalities [49]. Lung cancer is a significant reason for cancer-associated fatality [1], which is mainly categorized into small cell lung cancer (~15% cases) and non-small cell lung cancer (NSCLC, ~85% cases). The main histological types of NSCLC are adenocarcinoma and squamous cell carcinoma [50,51]. It has been suggested that early surgical resection of NSCLC can increase the 5-year survival by up to 70%; unfortunately, almost 75% of cases are detected at the time of advanced disease (Stages III/IV), making it difficult to manage the disease despite significant modern advancements in oncology practice [51]. Furthermore, past works have identified various diagnostic and prognostic signatures in cancers for patient stratification using gene expression and omics data; however, this approach has failed to capture the synergistic effects of gene expression [17,18,19]. Therefore, applying AI and MLT to omics data could be a promising approach for identifying and developing better prognostic and diagnostic models in cancers. The uses of AI in lung cancer detection have been a focus of research and gained significant scientific attention during the recent SARS-CoV-2 pandemic. The application of AI-based models using chest X-rays, CT scans, and PET scans have been suggested as important methods for detecting lung cancer [52,53]. Though the approach of image-based lung cancer detection is important for tackling this challenge, the use of molecular signatures can greatly surpass AI-based detection of the morphological changes, which become apparent after a long carcinogenic molecular transformation. 

A previous study identified 17 candidate genes in lung adenocarcinoma for predicting survival in non-smoking patients [54]. The study used weighted gene co-expression network analysis (WGCNA) and LASSO Cox regression to identify the prognostic signature; however, the model achieved an AUC-ROC of 0.736 on the training dataset [54]. Another study also used WGCNA and LASSO Cox regression, and identified four genes that predicted high and low overall survival in lung adenocarcinoma with an AUC-ROC of 0.71 on the training dataset [55]. Most of the previous studies focused on developing prognostic models for lung cancer; however, it is imperative to develop a model for predicting lung cancer with the high accuracy required for early detection and better management of patients. Furthermore, integrating the gene expression and interaction data has huge potential to identify the crucial genes associated with disease initiation. Therefore, this study implemented and identified the molecular transcriptome–interactome signatures for developing a LASSO-based machine learning model for predicting NSCLC. First, we identified the DEGs in lung cancer compared with normal samples using the lung-associated TCGA and GTEx data. Next, the human-specific interaction data were downloaded from BioGRID version 4.4.205, a continuously updated, large biomedical interaction repository currently holding almost 2.3 million proteins and genetic interactions from more than 78,000 publications [40]. The human interactions in BioGRID are also available with relevant literature references and therefore represent high-quality interaction data. In addition, the Cytoscape tool was used for reconstruction, visualization, and analysis of the biological network [41]. The important nodes from the DEGs’ interaction network were constructed on the basis of their degree, reflecting each node’s centrality in a particular interaction network. Hence, we identified the important nodes on the basis of their degree [56], indicating the identification of crucial nodes that may be involved in the proliferation of lung cancer. However, identifying the crucial genes that are relevant to detecting lung cancer is challenging. Therefore, we used the LASSO for feature selection and development of a model that identified a combined expression pattern of 17 genes (*KANK2, CLEC4D, ADRB2, CRYAB, NR4A1, CMTM5, ZBTB16, ACTC1, RAD51, KIF23, SYNE3, CLEC4E, CDKN2A, EGLN3, KIF14, RECQL4,* and *CDH1*) and their associated coefficients as a robust predictor of NSCLC. The performance of our developed LASSO model was highly accurate, with an AUC-ROC greater than 0.99 on most of the independent datasets of NSCLC, indicating that the selected 17-gene signature might be crucial for developing NSCLC (Figure 4). These genes belong to various categories, including tumor suppressors, oncogenes, translocated cancer genes, and transcription factors (Table S6). Furthermore, we validated our 17-gene signature across several studies and found most of these genes were differentially expressed; thus, our finding is supported by other studies (Table S10). 

Among the 17 signature genes, *KANK2, CLEC4D, ADRB2, CRYAB, NR4A1, CMTM5, ZBTB16, ACTC1,* and *SYNE3* showed downregulation in NSCLC with negative LASSO coefficients. Genes with negative coefficients indicate a lower risk of lung cancer if their expression is upregulated. *KANK2* gene encoding protein, also known as SRC interacting protein, is involved in transcription regulation and caspase-independent apoptosis. It is a tumor suppressor gene, and its downregulation is associated with NSCLC [57]. The mRNA expression level of *CLEC4D* was reported to be significantly lower in hepatocellular carcinoma [58]. According to GTEx V8 (https://gtexportal.org/, accessed on 15 October 2022), the lung is one of the tissues with high expression of *CLEC4D* mRNA. The gene *ADRB2* codes for the beta-2-adrenergic receptor, and its downregulation and polymorphisms are associated with lung cancer [59,60,61]. The alpha B-crystallin (encoded by *CRYAB*) is a molecular chaperon that binds to avert the aggregation of misfolded proteins and to inhibit apoptosis [62,63]. Studies have shown that the high expression of *CRYAB* is associated with tumor development and is a marker of poor prognosis for head and neck cancer [64], and breast cancer [65]. On the contrary, the role of *CRYAB* in lung cancer is controversial and needs more study [66]. *CMTM5* acts as a tumor-suppressor gene, and it is downregulated in several cancers, such as myeloid leukemia, ovarian cancer, prostate cancer, cervical carcinoma, and pancreatic cancer [67]. *ZBTB16* encodes for a zinc finger TF and is associated with the progression of the cell cycle. *ZBTB16* is underexpressed in multiple cancer types, including lung cancer [68]. Therefore, the selection and inclusion of downregulated genes with a negative coefficient in our LASSO model justified its high predictive accuracy, warranting further in vitro experiments to understand the mechanism(s) of NSCLC development. 

The *CDKN2A, EGLN3, KIF14, RECQL4,* and *CDH1* genes showed upregulation in NSCLC and had positive LASSO coefficients. Genes with positive coefficients increase the risk of lung cancer if their expression is upregulated. EGLN3 is a member of *Caenorhabditis elegans* gene *egl-9* (*EGLN*) family of oxygen- and α-ketoglutarate dependent prolyl hydroxylases. EGLN3 catalyzes the hydroxylation of extracellular signal-regulated kinase 3 (Erk3) and increased its stability, which is recognized as a strong, driver of cancers [69]. Thus, our finding has been validated by other studies where the *EGLN3* was reported to be vital for the growth of numerous cancers, including lung cancer [69]. The upregulated kinesin family member gene *KIF14* is a mitotic kinesin and plays an essential role in tumor development. Similar to lung cancer, overexpression of *KIF14* was also reported in several cancers, and the upregulation of this kinesin family member gene has been associated with poor prognosis [70]. RECQL4, a helicase known as a molecular motor, is involved in unwrapping the DNA, an essential event during DNA replication and DNA repair. Notably, the chromosomal site of the RECQL4 gene is considered as a hot-spot position for frequent mutation often highly detected in sporadic breast cancers [71]. Furthermore, Arora et al. demonstrated that the depletion of RECQL4 levels led to weakening of the DNA duplication rate and increased chemosensitivity in cultured breast cancer cells. Thus, their study confirmed that RECQL4 upregulation is linked with tumor progression in breast cancers [71]. Furthermore, another study showed that a high expression of the *BLM* gene, a paralog of *RECQL4*, was associated with poor prognosis in lung cancer [72]. Hence, we anticipate that further study of these genes in a model of a lung cancer cell line will eventually shed some more light on their involvement in NSCLC’s development and progression.

These feature genes are involved in several aspects of cancer progression as documented, and the important role of these targets in NSCLC indicates the importance of their detection by the LASSO model, which was also evident while performing a functional enrichment analysis of these target genes with DAVID Bioinformatics Resource 6.8. (Figure 5B) [73]. The functional overrepresentation analysis against the KEGG pathway database revealed that these targets are associated with cancer pathways. Moreover, analysis against Gene Ontology biological process terms indicated that these targets are involved in regulation of apoptosis, a crucial pathway dysregulated during cancer development. The inclusive picture involving the feature genes and their functional overrepresentation analyses revealed the importance of these factors in developing the MLT-based model. 

The LASSO-based model has been used to diagnose other diseases, indicating its potential for detecting cancers [74,75]. As noted previously, early cancer detection is key to preventing several cancer-associated complications. In addition, this can also reduce the significant economic burden on the healthcare system by reducing the chance of metastasis and mortality. Our study used a systems biology and LASSO-based approach, and identified the transcriptome–interactome signatures that achieved high accuracy in predicting NSCLC. Thus, evidence of the high accuracy of our model indicated that the strategy of integrating transcriptome–interactome signatures has enormous potential to develop better models for predicting other diseases, including various cancers. In addition to the late diagnosis, other obstacles preventing the long-term survival of NSCLC patients include a lack of advanced treatment and an accurate prognosis model due to the disease’s heterogeneity, as well as differences in cancer care facilities across the world.

Our study has a few limitations, including the following. Firstly, the publicly available data are imbalanced and contain many NSCLC cancer samples compared with normal samples. Therefore, we used the AUC-ROC and AUC-PRC curves to check the performance of the model developed on imbalanced data at different threshold values. Second, our model did not include gene mutations, intra-tumoral heterogeneity, and other clinical features associated with cancer. Third, we identified a 17-gene signature that needs to be further validated using qRT-PCR in several clinical samples of NSCLC. Finally, tissue biopsies are needed to quantify the genes’ expression levels, which are invasive, costly, and time-consuming.

## 5. Conclusions

In summary, we conducted an integrative approach to identify the transcriptome and interactome signatures for discriminating NSCLC from normal samples. We then applied LASSO logistic regression to find a 17-gene signature and developed a model for predicting NSCLC. The performance of our model showed high accuracy across several independent datasets of NSCLC. Finally, we developed a web application, *NSCLCpred* (https://hifzuransari.shinyapps.io/NSCLCpred/, accessed on 31 October 2022), for detecting NSCLC using the expression profile of 17 genes. Our findings could be helpful in creating a new strategy for diagnosing NSCLC patients. Furthermore, we expect our identified gene signature to provide novel insights and therapeutic targets for NSCLC. 

## Figures and Tables

**Table 1:** The number of TCGA and GTEx lung samples used for DEGs analysis and development of the LASSO logistic regression model.

Sample Type	Disease Class	Training Dataset (TR)	Test Dataset 1 (TD1)
Primary tumor	Lung cancer (positive = 1)	802	209
Recurrent tumor		2	0
Normal solid tissue	Normal lung (negative = 0)	91	18
Normal tissue (GTEx)		233	55
Total		1128	282

**Table 2:** The top 20 upregulated and 20 downregulated hub genes from the DEGs’ interaction network.

Upregulate Genes			Downregulated Genes		
Log2FC	Degree	Name	Log2FC	Degree	Name
4.76	698	SOX2	−5.16	279	GPR17
4.33	804	CDC20	−5.06	297	ZBTB16
4.19	1143	ANLN	−4.13	278	CMTM5
4.08	1063	KIF20A	−3.66	723	ACTC1
3.73	1834	KIF14	−3.55	294	USHBP1
3.51	817	AURKB	−2.87	429	TRIM63
3.29	550	MKI67	−2.84	404	ADRB2
3.24	635	CDK1	−2.82	715	LRRK2
3.14	577	RAD51	−2.70	270	NR4A1
3.12	1207	MCM2	−2.70	764	MEOX2
3.10	695	PLK1	−2.69	843	CAV1
2.86	529	CDKN2A	−2.59	315	CLEC4D
2.62	1032	KIF23	−2.46	411	CLEC4E
2.58	934	ECT2	−2.43	455	GPR182
2.50	986	PRC1	−2.41	433	SYNE3
2.50	1465	RECQL4	−2.33	342	CRYAB
2.37	553	KRT31	−2.27	294	KANK2
2.35	1354	EGLN3	−2.19	297	ALB
2.31	849	CDH1	−2.09	367	LMO2
2.14	1189	AGR2	−2.07	348	HECW2

**Table 3:** Performance of the LASSO model on the test dataset TD1.

Threshold	Accuracy	Specificity	Sensitivity	TN	TP	FN	FP	NPV	PPV
0	0.741	0.000	1.000	0	209	0	73	NA	0.741
0.1	0.982	0.945	0.995	69	208	1	4	0.986	0.981
0.2	0.982	0.945	0.995	69	208	1	4	0.986	0.981
0.3	0.982	0.945	0.995	69	208	1	4	0.986	0.981
0.4	0.982	0.945	0.995	69	208	1	4	0.986	0.981
**0.5**	**0.986**	**0.959**	**0.995**	**70**	**208**	**1**	**3**	**0.986**	**0.986**
0.6	0.982	0.959	0.990	70	207	2	3	0.972	0.986
0.7	0.986	0.973	0.990	71	207	2	2	0.973	0.990
0.8	0.982	0.973	0.986	71	206	3	2	0.959	0.990
0.9	0.986	1.000	0.981	73	205	4	0	0.948	1.000
1	0.259	1.000	0.000	73	0	209	0	0.259	NA

NA means not available.

## Data Availability

The computer code, LASSO Model, and supporting data of *NSCLCpred* are available at the GitHub repository (https://github.com/firozimtech/NSCLCpred, accessed on 31 October 2022). The R/Shiny web application of *NSCLCpred* is available at https://hifzuransari.shinyapps.io/NSCLCpred/ (accessed on 31 October 2022).

## Supplementary Materials

The following supporting information can be downloaded at https://www.mdpi.com/article/10.3390/biology11121752/s1. Figure S1: Performance of logistic regression models on the test dataset 1 (TD1). Table S1: The number of samples in the test dataset 2 (TD2) used to assess the performance of the LASSO model. Table S2: List of the top 20 DEGs based on log2FC values. Table S3: Upregulation of genes in lung cancer compared with normal samples. A gene was considered to be upregulated at log2FC > 2 and adj.p.value < 0.001. Table S4: Downregulation of genes in lung cancer compared with normal samples. A gene was considered to be downregulated at log2FC < −2 and adj.p.value < 0.001. Table S5: Genes with non-zero coefficients selected by using LASSO. Table S6: The 17-gene signature of the LASSO model is associated with different gene families according to the Molecular Signature Database (MSigDB). Table S7: Performance of the LASSO model on the independent TD2 dataset GSE18842. Table S8: Performance of the LASSO model on the independent TD2 dataset GSE27262. Table S9: Performance of the LASSO model on the independent TD2 dataset GSE19804. Table S10: Expression patterns of the 17-gene signature across various studies on lung cancer. The data were extracted from Expression Atlas release 38 (https://www.ebi.ac.uk/gxa/home, accessed on 7 August 2022).
