# Supplementary material for: A Systems Biology and LASSO-Based Approach to Decipher the Transcriptome–Interactome Signature for Predicting Non-Small Cell Lung Cancer
Source: Biology (Basel). 2022 Nov 30;11(12):1752. doi: 10.3390/biology11121752 (PMC9774707; doi:10.3390/biology11121752)
Supplement: Supplementary file 1 [file biology-11-01752-s001.zip › Supplementary_12Dec2022/Table_S1_S2_S5-S9.pdf]

Table S1: The number of samples in the test dataset 2 (TD2) used to assess the performance of the LASSO model.

GEO accession	Lung cancer sample	Adjacent non-tumor sample
GSE18842	46	45
GSE27262	25	25
GSE19804	60	60
**Total number of samples**	**131**	**130**

Table S2: List of the top 20 DEGs based on log2FC values.

**Upregulate genes**				**Downregulated genes**			
**Gene**	log2FC	p.value	adj.p.val	Gene	log2FC	p.value	adj.p.val
CST1	7.58	8.36E-274	6.51E-272	*DEFA1B*	-7.38	0	0
FAM83A	6.70	2.38E-290	2.52E-288	*SLC6A4*	-6.98	0	0
KRT16	6.69	2.97E-141	2.90E-140	*DEFA1*	-6.93	0	0
MMP13	6.69	9.43E-215	2.74E-213	*SFTPC*	-6.81	4.12E-129	3.34E-128
MMP12	6.40	3.53E-275	2.82E-273	*CA4*	-6.63	0	0
CA9	6.33	2.56E-226	9.01E-225	*DEFA3*	-6.62	7.17E-296	8.27E-294
TMPRSS11E	6.33	5.03E-219	1.56E-217	*CD300LG*	-6.57	0	0
AKR1B10	6.30	1.67E-120	1.19E-119	*FCN3*	-6.32	0	0
PRAME	6.30	1.08E-155	1.31E-154	*IL1RL1*	-6.26	0	0
TMPRSS4	6.26	2.19E-300	2.79E-298	*ADAMTS7P3*	-6.19	0	0
GPR87	6.25	2.93E-180	5.20E-179	*ADH1B*	-6.18	6.75E-226	2.35E-224
RP11-40C6.2	6.08	8.95E-287	8.83E-285	*RP11-286H15.1*	-6.16	0	0
PITX1	5.98	7.18E-229	2.62E-227	*ADRA1A*	-6.11	0	0
AP000349.2	5.91	2.90E-270	2.11E-268	*ADAMTS8*	-6.03	0	0
SERPINB5	5.90	9.51E-124	7.10E-123	*TMEM100*	-6.02	0	0
GJB2	5.83	2.27E-267	1.57E-265	*GRIA1*	-6.02	9.82E-305	1.42E-302
RP5-940J5.9	5.83	7.68E-118	5.25E-117	*CLDN18*	-5.96	3.76E-177	6.33E-176
MMP11	5.77	0	0	*MYOC*	-5.90	0	0
CASC9	5.67	1.11E-157	1.40E-156	*BTNL9*	-5.86	0	0
CYP24A1	5.66	3.31E-173	5.25E-172	*AGER*	-5.78	2.54E-288	2.59E-286

Table S5: Genes with non-zero coefficients selected by using LASSO.

Gene	coefficient	log2FC
Intercept	-3.207	
KANK2	-1.016	-2.27
CLEC4D	-0.929	-2.59
ADRB2	-0.64	-2.84
CRYAB	-0.533	-2.33
NR4A1	-0.322	-2.7
CMTM5	-0.297	-4.13
ZBTB16	-0.174	-5.06
ACTC1	-0.12	-3.66
RAD51	-0.118	3.14
KIF23	-0.117	2.62
SYNE3	-0.087	-2.41
CLEC4E	0.136	-2.46
CDKN2A	0.403	2.86
EGLN3	0.459	2.35
KIF14	0.675	3.73
RECQL4	1.372	2.5
CDH1	1.457	2.31

Table S6: The 17-gene signature of the LASSO model is associated with different gene families according to the Molecular Signature Database (MSigDB).

	cytokines and growth factors	transcription factors	homeodomain proteins	cell differentiation markers	protein kinases	translocated cancer genes	oncogenes	tumor suppressors
tumor suppressors	0	0	0	CDH1	0	0	0	RECQL4, CDKN2A, CDH1
oncogenes	0	ZBTB16	0	0	0	ZBTB16	ZBTB16	
translocated cancer genes	0	ZBTB16	0	0	0	ZBTB16		
protein kinases	0	0	0	0	0			
cell differentiation markers	0	0	0	CDH1				
homeodomain proteins	0	0	0					
transcription factors	0	NR4A1, ZBTB16						
cytokines and growth factors	CMTM5							

Table S7: Performance of the LASSO model on the independent TD2 dataset GSE18842.

Threshold	Accuracy	Specificity	Sensitivity	TN	TP	FN	FP	NPV	PPV
0	0.505	0	1.000	0	46	0	45	NA	0.505
0.1	1.000	1	1.000	45	46	0	0	1.000	1.000
0.2	1.000	1	1.000	45	46	0	0	1.000	1.000
0.3	1.000	1	1.000	45	46	0	0	1.000	1.000
0.4	1.000	1	1.000	45	46	0	0	1.000	1.000
**0.5**	**1.000**	**1**	**1.000**	**45**	**46**	**0**	**0**	**1.000**	**1.000**
0.6	0.978	1	0.957	45	44	2	0	0.957	1.000
0.7	0.967	1	0.935	45	43	3	0	0.938	1.000
0.8	0.934	1	0.870	45	40	6	0	0.882	1.000
0.9	0.901	1	0.804	45	37	9	0	0.833	1.000
1	0.495	1	0.000	45	0	46	0	0.495	NA

Table S8: Performance of the LASSO model on the independent TD2 dataset GSE27262.

Threshold	Accuracy	Specificity	Sensitivity	TN	TP	FN	FP	NPV	PPV
0	0.500	0	1.000	0	25	0	25	NA	0.500
0.1	1.000	1	1.000	25	25	0	0	1.000	1.000
0.2	1.000	1	1.000	25	25	0	0	1.000	1.000
0.3	1.000	1	1.000	25	25	0	0	1.000	1.000
**0.4**	**1.000**	**1**	**1.000**	**25**	**25**	**0**	**0**	**1.000**	**1.000**
**0.5**	**0.980**	**1**	**0.960**	**25**	**24**	**1**	**0**	**0.962**	**1.000**
0.6	0.980	1	0.960	25	24	1	0	0.962	1.000
0.7	0.920	1	0.840	25	21	4	0	0.862	1.000
0.8	0.920	1	0.840	25	21	4	0	0.862	1.000
0.9	0.860	1	0.720	25	18	7	0	0.781	1.000
1	0.500	1	0.000	25	0	25	0	0.500	NA

Table S9: Performance of the LASSO model on the independent TD2 dataset GSE19804.

Threshold	Accuracy	Specificity	Sensitivity	TN	TP	FN	FP	NPV	PPV
0	0.500	0.000	1.000	0	60	0	60	NA	0.500
0.1	0.808	0.983	0.633	59	38	22	1	0.728	0.974
0.2	0.792	1.000	0.583	60	35	25	0	0.706	1.000
0.3	0.742	1.000	0.483	60	29	31	0	0.659	1.000
0.4	0.725	1.000	0.450	60	27	33	0	0.645	1.000
**0.5**	**0.725**	**1.000**	**0.450**	**60**	**27**	**33**	**0**	**0.645**	**1.000**
0.6	0.725	1.000	0.450	60	27	33	0	0.645	1.000
0.7	0.717	1.000	0.433	60	26	34	0	0.638	1.000
0.8	0.658	1.000	0.317	60	19	41	0	0.594	1.000
0.9	0.625	1.000	0.250	60	15	45	0	0.571	1.000
1	0.500	1.000	0.000	60	0	60	0	0.500	NA
