# Supplementary figures and images for: A Systems Biology and LASSO-Based Approach to Decipher the Transcriptome–Interactome Signature for Predicting Non-Small Cell Lung Cancer
Source: Biology (Basel). 2022 Nov 30;11(12):1752. doi: 10.3390/biology11121752 (PMC9774707; doi:10.3390/biology11121752)

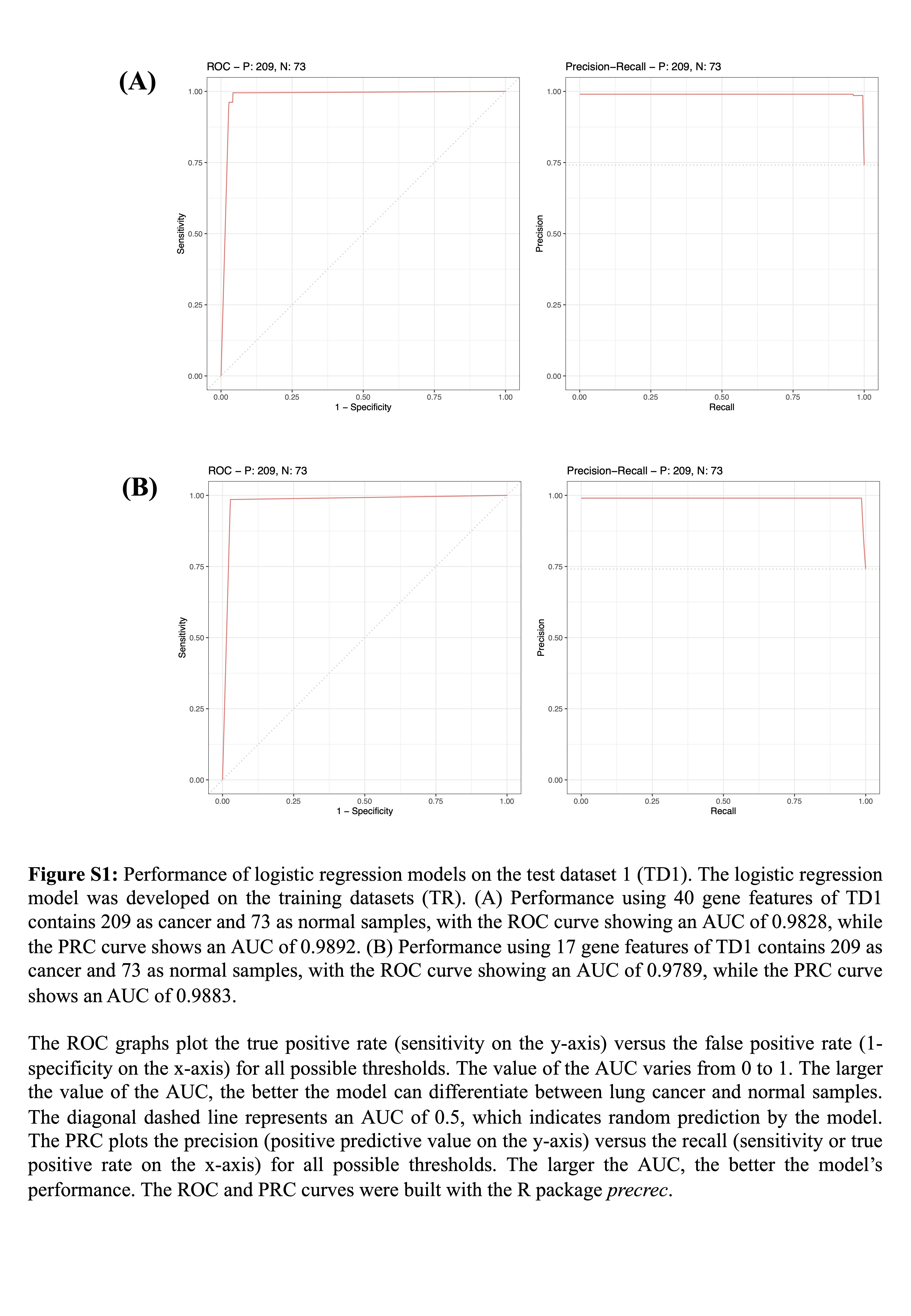

Supplement: Supplementary file 1 [file biology-11-01752-s001.zip › Supplementary_12Dec2022/Figure_S1.jpg]
